# Laparoscopic sacrocolpopexy for neovaginal prolapse in a patient after male-to-female sex reassignment surgery

**DOI:** 10.1590/S1677-5538.IBJU.2018.0086

**Published:** 2019-07-27

**Authors:** Marek Roslan, Marcin Markuszewski, Wojciech Piaskowski, Wojciech Połom, Sławomir Letkiewicz

**Affiliations:** 1 Department of Urology, Faculty of Medicine, University of Warmia and Mazury, Olsztyn, Poland; 2 Department of Urology, Medical University of Gdańsk, Gdańsk, Poland; 3 Institute of Immunology and experimental therapy, Polish Academy of Science, Wrocław, Poland

## Abstract

**Introduction:**

Male / female sex reassignment surgery is performed on transsexuals, and includes removal of the male external genitalia, and creation of the neovagina from the skin of the penis, usually allowing sexual intercourse (1, 2). The incidence of the prolapse of the neovagina is not known; however, such complication is observed relatively rarely (3, 4). the long-term outcomes of prolapse treatment in transsexual patients are not available in the literature. The purpose of this study was to demonstrate laparoscopic sacrocolpopexy to repair a neovagina prolapse in a patient after male-to-female sex reassignment surgery.

**Materials and Methods:**

In september 2013, a laparoscopic repair was performed on a 44-year-old woman who presented a neovaginal prolapse of pelvic organ prolapse quantification (pop-q) stage iii, twenty one years after sex reassignment surgery. This condition caused painful or even indisposed intercourse. in may 2013, the patient underwent unsuccessful vaginal treatment with the suturing device. Before the initial surgery, the patient was examined with cystoscopy, urodynamics and microbiology; no pathologies were found. laparoscopic repair of the neovaginal prolapse followed the principles described previously in the natural female (5). In the supine lithotomy position, a standard multiport laparoscopic sacrocolpopexy was performed with the use of the polypropylene mesh (Artisyn^®^ y-shaped mesh, ethicon, inc somerville, nj.) and coated polyglactin sutures.

**The following steps were applied:**

exposure of the anterior and posterior neovaginal walls; suturing the bifurcated end of the mesh to the neovagina; longitudinal incision of the parietal peritoneum and creation of a tunnel for the mesh; fixation of the proximal end of the mesh to the promontorium; and closure of the parietal peritoneum over the mesh that was placed retroperitoneally. The draining tube was left for 24 hours.

**Results:**

The operation was completed successfully, with no blood loss or complications. The operative time was 115 minutes. The patient was discharged on the 2nd postoperative day. In a four-year follow-up, the patient presented significant improvement of symptoms, a small prolapse of approximate pop-q stage i, and declared performing satisfying intercourse.

**Conclusions:**

Laparoscopic sacrocolpopexy with the use of a polypropylene mesh to repair a neovaginal prolapse in transsexuals seems to be a valuable alternative to other procedures. Further observations and evaluation of a greater number of patients will be necessary to assess the actual value of the method.

## ARTICLE INFO

**Available at: http://www.intbrazjurol.com.br/video-section/20180086_Roslan_et_al**

**Int Braz J Urol. 2019; 45 (video #13): 643-4**

